# Worldwide research trends on the tumor immunometabolism–cancer immunotherapy nexus: a systematic bibliometric review

**DOI:** 10.3389/fonc.2026.1962391

**Published:** 2026-10-19

**Authors:** Jingshuai Huang, Haiyuan Zhong, Liwei Li, Xiaoping Wang, Yuling Lai, Jincheng Li, Dan Jiang, Mingyu Lai

**Affiliations:** 1 Department of Gastroenterology, The First Affiliated Hospital of Guangxi Medical University, Nanning, China; 2 Department of Gastroenterology, The Second Affiliated Hospital of Guangxi Medical University, Nanning, China

**Keywords:** bibliometrics, cancer immunotherapy, CAR-T cells, immune checkpoint inhibitors, immunometabolism, metabolic reprogramming, tumor microenvironment

## Abstract

**Background:**

Metabolic competition, nutrient restriction and metabolite accumulation in the tumor microenvironment reshape immune-cell fate and can constrain immune checkpoint blockade and adoptive cell therapies. We systematically mapped whether the tumor immunometabolism literature has shifted toward immunotherapy-oriented translation.

**Methods:**

We conducted a systematic bibliometric mapping review reported with reference to PRI SMA 2020. Web of Science Core Collection and Scopus were searched on 26 July 2026 for English-language journal articles and reviews published from 2001 to 2025 that linked cancer, immune-cell metabolism and immunotherapy. Prespecified metadata eligibility criteria were applied independently to each database. Web of Science was the primary dataset and Scopus provided parallel validation. Bibliometrix, VO Sviewer and Cite Space characterized publication growth, contributors, citation structure and keyword evolution. Between-group and stratum comparisons used non-parametric tests, with test statistics, effect sizes and Benjamini–Hochberg-adjusted P values reported for every comparison.

**Results:**

We retained 3,214 Web of Science and 3,513 Scopus records. Publications from 2020–2025 represented 83.7% and 83.0% of the respective datasets, reproducing the recent expansion across databases. China contributed the most Web of Science publications (n=1,470), whereas the United States had more total citations (70,049 vs 55,911) and a higher mean citation rate (96.4 vs 38.0 citations per article). Articles involved larger teams than reviews (median 10 vs 4 authors), whereas reviews cited more references (median 136 vs 50; both adjusted p<0.001). Leading keywords included immunotherapy (n=768), tumor microenvironment (n=469), metabolism (n=198), T cells (n=187), immunometabolism (n=155), hypoxia (n=149), metabolic reprogramming (n=133), immune checkpoint inhibitors (n=121) and CAR-T cells (n=101). Highly cited and citation-burst literature progressed from hypoxia, adenosine and metabolic competition toward T-cell exhaustion, mitochondrial fitness, regulatory T-cell adaptation and metabolically engineered cellular therapy.

**Conclusion:**

This systematic bibliometric review identifies a reproducible shift toward metabolism-informed checkpoint and cellular-therapy strategies, but bibliometric proximity to clinical terminology should not be interpreted as evidence of clinical efficacy or readiness.

## 1 Introduction

Cancer immunotherapy has changed the treatment landscape for multiple malignancies, yet durable benefit remains restricted to a subset of patients (1, 2). One reason is that antitumor immunity operates within a metabolically hostile tumor microenvironment. Rapidly proliferating malignant cells consume glucose, amino acids and oxygen, while producing lactate, adenosine and other metabolites that alter immune-cell activation, differentiation and persistence (2–4). These constraints do not simply accompany immune dysfunction; influential experimental studies demonstrated that metabolic competition and specific metabolic checkpoints can directly shape T-cell activity and tumor progression (5, 6).

Immune checkpoints and cellular therapies are themselves metabolically active interventions. Programmed cell death protein 1 signaling suppresses glycolytic reprogramming and promotes fatty-acid oxidation in T cells (7), while hypoxia-inducible factor signaling links oxygen deprivation to PD-L1 expression and myeloid-mediated immune suppression (8). Costimulatory design can determine the metabolic program and memory development of chimeric antigen receptor (CAR)-T cells (9). Lipid handling, glutamine use, mitochondrial fitness and pyruvate transport have consequently emerged as manipulable determinants of antitumor function (10–13).

The field has therefore expanded beyond a mechanism-centered account of immune-cell metabolism (14–16). It now includes metabolic biomarkers, combination therapies, engineered cell products and interventions intended to overcome checkpoint resistance or improve cellular persistence. However, the literature is dispersed across immunology, oncology, metabolism and bioengineering. Conventional narrative reviews describe individual pathways in depth, but they do not quantify when the field expanded, which countries and institutions shaped it, how its collaboration structure differs from its citation impact, or whether its conceptual vocabulary has moved toward clinically oriented immunotherapy.

Bibliometric analysis can address these field-scale questions by combining publication trends, citation structure, collaboration networks and the temporal evolution of keywords. It is particularly useful here because “translation” is not a single outcome: it may be reflected in the increasing proximity of mechanistic work to immune checkpoint inhibitors, CAR-T cells, treatment resistance and patient-relevant cancer contexts, while still falling short of direct evidence from clinical trials.

This systematic bibliometric mapping review examined the tumor immunometabolism–cancer immunotherapy nexus from 2001 to 2025. Web of Science was used for the primary analysis and Scopus for cross-database validation. We assessed publication growth, document characteristics, geographic and institutional leadership, journal and author influence, citation bursts and keyword evolution. We then integrated these layers to determine whether the field had progressed from describing metabolic immune suppression toward metabolism-informed checkpoint and cellular-therapy strategies. This design maps a change in research orientation; it does not treat bibliometric proximity to clinical language as evidence of patient benefit.

## 2 Materials and methods

### 2.1 Review design and reporting framework

This study was designed as a systematic bibliometric mapping review and is reported with reference to the PRI SMA 2020 statement (17), adapted to the analysis of bibliographic records rather than intervention-effect estimates. The review question was: how has worldwide research linking tumor immunometabolism with cancer immunotherapy evolved in volume, contributors, collaboration, citation structure and thematic orientation from 2001 to 2025? The review was not prospectively registered, and no separate protocol was published.

Eligibility was specified using an adapted PICO S framework. The population comprised bibliographic records concerning malignant disease; the intervention or exposure domain comprised immune-cell metabolic processes in an immunotherapy context; no comparator was required; outcomes were bibliometric indicators of growth, contribution, collaboration, citation influence and thematic evolution; and eligible study designs were English-language journal Articles or Reviews published from 2001 through 2025. Records outside the publication window, non-English records, non-journal source types and document types other than Articles or Reviews were excluded. Because this review mapped a research field rather than estimating treatment effects, patients, clinical interventions and comparative effect measures were not units of synthesis.

### 2.2 Data sources, search strategy and record selection

Web of Science Core Collection and Scopus were searched on 26 July 2026. Search terms combined three concept blocks: malignancy; immunometabolism or metabolic processes in immune-cell contexts; and immunotherapy, including immune checkpoint blockade, PD-1/PD-L1/CTLA-4-directed therapy, CAR-T/TCR-T cells and adoptive cell therapy. Web of Science used topic-field searching with NEAR/5 proximity operators, whereas Scopus used TITLE-AB S-KEY searching with W/5 proximity operators. The complete database-specific search strings, limits and search date are provided in **Supplementary Methods**. No unpublished material, submitted manuscripts or personal communications were eligible.

Prespecified eligibility rules were applied to the exported metadata. The Web of Science search returned 3,386 records; 164 records with document types other than Article or Review and eight non-English records were excluded, leaving 3,214 records. The Scopus search returned 3,602 records; 79 non-English records and ten records with non-journal source types were excluded, leaving 3,513 records. Web of Science and Scopus were analyzed as independent corpora rather than pooled; cross-database deduplication was therefore not used to determine a combined sample size. The adapted PRI SMA flow diagram reports each database stream separately (**Figure 1**).

**Figure 1:**
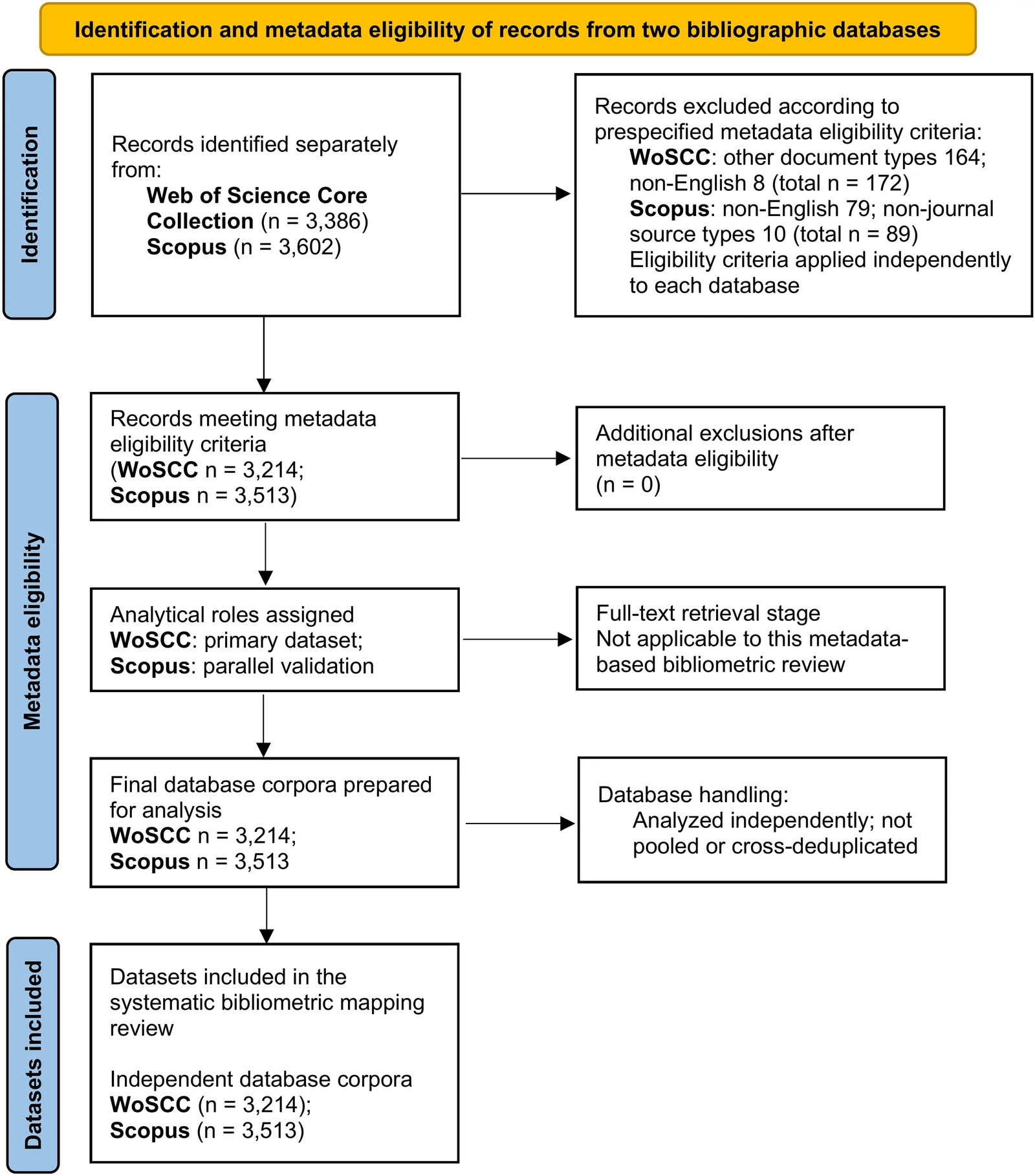
PRISMA 2020 flow diagram adapted for parallel bibliometric datasets. Records were identified in Web of Science Core Collection (Wo S) and Scopus on 26 July 2026 for publications from 2001 to 2025. Prespecified metadata eligibility rules were applied to each database separately: English-language journal Articles or Reviews only. Wo S: 3,386 records identified; 164 records of other document types and 8 non-English records excluded; 3,214 records retained for the primary analysis. Scopus: 3,602 records identified; 79 non-English records and 10 records from non-journal sources excluded; 3,513 records retained for parallel validation. The two databases were analyzed independently and were not pooled, so cross-database deduplication was not performed.

### 2.3 Data extraction, data items and bias considerations

For eligible records, the extracted bibliographic fields included publication year, document type, authors, affiliations, corresponding-author country, journal, author keywords, cited references, citation counts and reference counts when available. Journal impact factor and quartile were verified against the 2025 Journal Citation Reports and used only as descriptive journal-level attributes. Data were processed at the record level; no patient-level data or unpublished study results were collected.

Country-level analyses were based on the corresponding author’s country, that is, the country of the corresponding author’s address as indexed in the source database. Each record was assigned to exactly one country; when a corresponding author listed addresses in more than one country, the first listed corresponding-author address was used. Regional entries for Hong Kong SAR, Macao SAR and Taiwan Province of China were aggregated into China. Country output was therefore counted by corresponding-author country and not by a full count of all author affiliations. Institution-level analyses used a separate document-level rule: each publication contributed at most one count to a given institution, even when several authors on the same paper shared that affiliation.

Conventional study-level risk-of-bias tools for intervention reviews were not applicable because this review did not synthesize clinical effect estimates or make judgments about treatment efficacy. Potential bibliometric biases were addressed by reporting the complete database-specific search strings, applying explicit metadata eligibility rules, analyzing Web of Science and Scopus independently, normalizing author, affiliation and keyword variants, and interpreting citation and journal metrics as indicators of visibility rather than methodological quality. Residual risks from database coverage, English-language restriction, indexing delay, citation-age effects and name disambiguation are reported in the Discussion.

Publications were classified by international collaboration status following the standard bibliometric convention. A single-country publication (SCP) is a publication in which all authors, including the corresponding author, are affiliated with institutions in a single country. A multi-country publication (MCP) is a publication with author affiliations in more than one country. MCP proportion is the number of multi-country publications divided by the total number of publications assigned to that country, expressed as a percentage (MCP %). Because country assignment is by corresponding author, an MCP is a publication whose corresponding-author country differs from the country of at least one other author’s affiliation.

### 2.4 Bibliometric analysis

Web of Science was treated as the primary analytical dataset. Scopus was analyzed independently to assess the database robustness of publication trends, document-type and authorship patterns, geographic and institutional contributions, journal distribution, author productivity and keyword evolution. These cross-database comparisons are reported in **Supplementary Figure 1**-**6**. Reference citation bursts and the detailed VO Sviewer network interpretations were based on the Web of Science dataset unless otherwise stated. Metadata were processed in R 4.6.1 with bibliometrix and supporting packages (18). The journal impact factor (JIF) and quartile were verified against the 2025 edition of the Journal Citation Reports (JCR) via the official website. Journal-level impact factor was used only as a descriptive stratifier and not as a proxy for the methodological quality of individual studies.

VO Sviewer 1.6.21 was used for co-authorship and author-keyword co-occurrence networks with full counting and association-strength normalization (19). For the Web of Science keyword map, a minimum of 25 occurrences was required; 64 of 4,566 author keywords met the threshold. The resulting map contained 64 items, six clusters, 956 links and a total link strength of 3,797. Author maps used a minimum of ten documents per author, excluded documents with more than 25 authors, and retained 27 authors. Organization and country maps used minimum thresholds of 25 and five documents, respectively. No nodes were manually selected to alter the keyword map.

Cite Space 7.0 was used for reference and keyword citation-burst detection (20). Burst intervals were interpreted as temporal signals of rapidly increasing attention. Automatically generated keyword cluster labels were not used as confirmatory evidence because the supplied cluster output reported silhouette values of zero; cluster-level interpretation therefore relied on the VO Sviewer map and normalized keyword data instead.

Author names, affiliations and keyword variants were normalized before aggregation. A documented thesaurus merged variants such as tumour/tumor microenvironment, CAR T/CAR-T cells and singular/plural immune checkpoint inhibitor terms. Common-name authors were checked against record-level affiliations where possible. Dataset-level H indices refer only to publications included in this corpus.

### 2.5 Statistical analysis

Publication and reference-count variables were non-normally distributed and are reported as medians with interquartile ranges (IQRs). Two-group comparisons used two-sided Wilcoxon rank-sum tests, reported with the Wilcoxon rank-sum statistic *W*, the two-sided P value from the normal approximation with continuity correction, and the rank-biserial correlation *r*_rb_ as the effect-size estimate (*r*_rb_ = 2*W*/(n_1_n_2_) − 1; positive values indicate higher values in the first-named group). Comparisons across the three journal-impact-factor strata (0–10, 10–20 and >20) used Kruskal–Wallis omnibus tests, reported with the test statistic *H* and its degrees of freedom, followed by pairwise two-sided Wilcoxon rank-sum tests. Effect sizes are reported as *r*_rb_ for two-group and pairwise comparisons and as ϵ^2^ = *H*/((n^2^ − 1)/(n + 1)) for the Kruskal–Wallis omnibus tests. Following conventional benchmarks, |*r*_rb_| or ϵ^2^ of 0.10, 0.30 and 0.50 were interpreted as small, medium and large; the same thresholds were applied to ϵ^2^, which we interpret as small throughout. Two-sided adjusted *p* < 0.05 was considered statistically significant.

Multiplicity was controlled using the Benjamini–Hochberg (BH) procedure within prespecified correction families, applied at a false-discovery rate of 0.05. Family 1 comprised the two document-type comparisons (Articles vs Reviews) for the number of references and the number of authors. Family 2 comprised the pairwise comparisons among journal-impact-factor strata, tested separately for each document type and each available outcome, giving 12 pairwise tests in total across the two databases. Family 1 and Family 2 were corrected independently; no comparison appeared in more than one family. Omnibus Kruskal–Wallis tests were interpreted as hypothesis-generating and were not included in either BH family. Because sample sizes are large, effect sizes are reported alongside all P values, and P values are interpreted only together with their corresponding effect size. Statistical test statistics, effect sizes and exact and Benjamini-Hochberg-adjusted P values for every comparison, together with the composition of each correction family, are reported in **Supplementary Table 1**.

## 3 Results

### 3.1 Study retrieval and screening

The metadata-based eligibility process retained 3,214 of 3,386 Web of Science records and 3,513 of 3,602 Scopus records (**Figure 1**). Web of Science provided the primary sample for detailed bibliometric, citation and statistical analyses. Scopus yielded a slightly larger eligible set and was analyzed separately for cross-database comparison of publication, authorship, geographic, journal, author and keyword patterns (**Supplementary Figure 1**–**6**). The two datasets were not combined because differences in indexing, document identifiers and affiliation structures would otherwise introduce duplicate and source-dependent records.

### 3.2 Global publication trends

Web of Science indexed only four eligible papers in 2002 and 44 cumulatively by 2009. Annual production increased to 42 papers in 2015, 125 in 2019, 245 in 2020 and 774 in 2025 (**Figure 2A**). The 2,690 publications from 2020–2025 represented 83.7% of the Web of Science corpus. The corresponding Scopus period contained 2,916 publications, or 83.0% of its eligible records, and annual production reached 826 in 2025. Scopus therefore reproduced the overall temporal pattern and supported the Web of Science-based assessment of recent global publication growth (**Supplementary Figure 1**).

**Figure 2:**
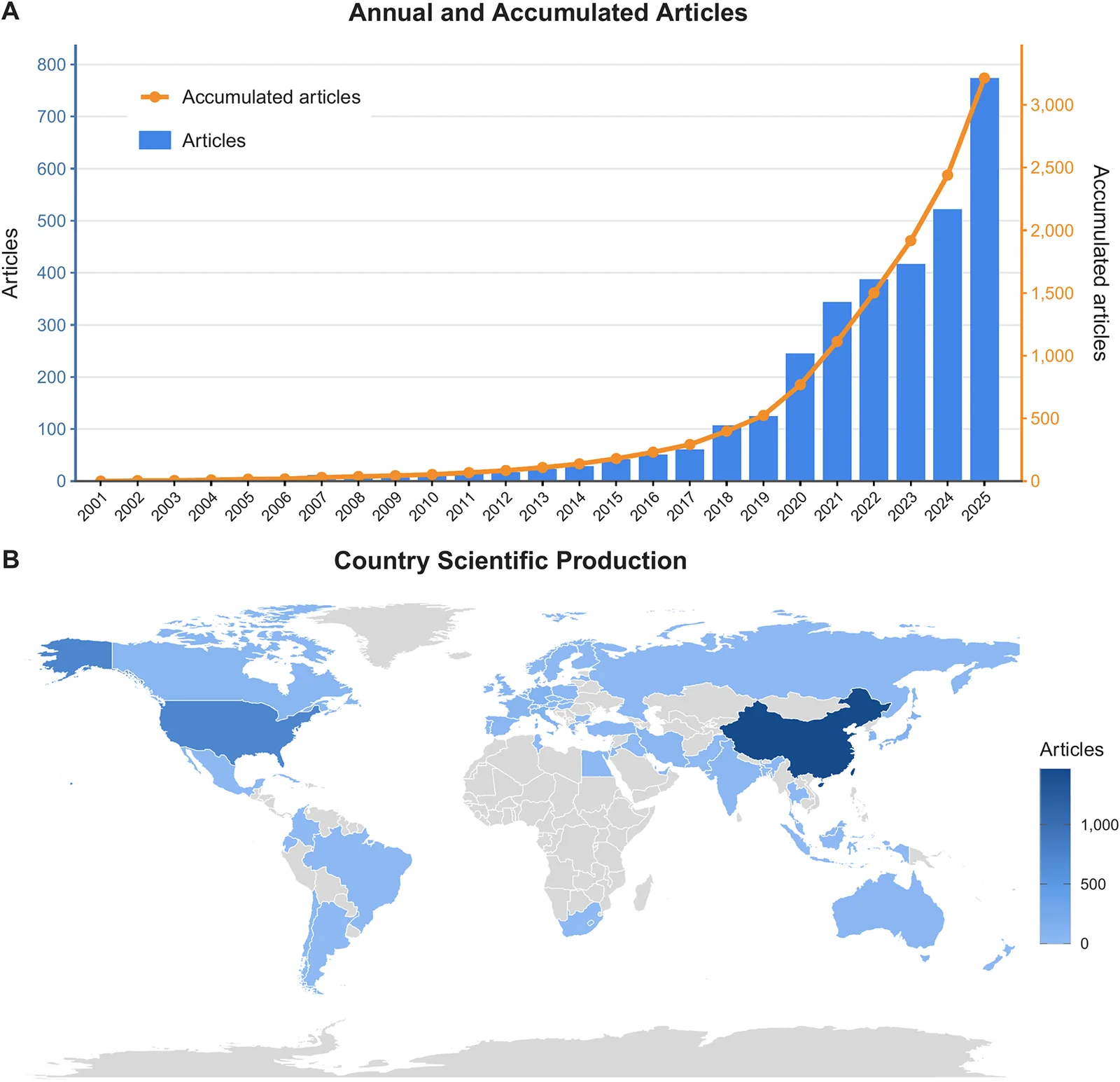
Annual publication growth and geographic distribution (Wos, 2001-2025). **(A)** Annual (bars) and cumulative (line) number of publications per year; a single shared y-axis is used for both. **(B)** World map of scientific production, colored by the number of publications whose corresponding author was affiliated in each country; each record contributes a single count, darker blue indicates more publications, and gray indicates countries with no eligible records. Affiliations from Hong Kong SAR, Macao SAR and Taiwan Province of China were aggregated under China in this analysis. Production accelerated after 2020 and was concentrated in China and the United States.

The document-type composition also changed in scale. In the harmonized Web of Science classification, 2,175 records were Articles and 1,039 were Reviews. Both types increased sharply after 2020; by 2025, annual output reached 504 Articles and 270 Reviews (**Figure 3A**). The large review component indicates sustained demand for conceptual synthesis in a field whose mechanisms and therapeutic applications are expanding simultaneously.

**Figure 3:**
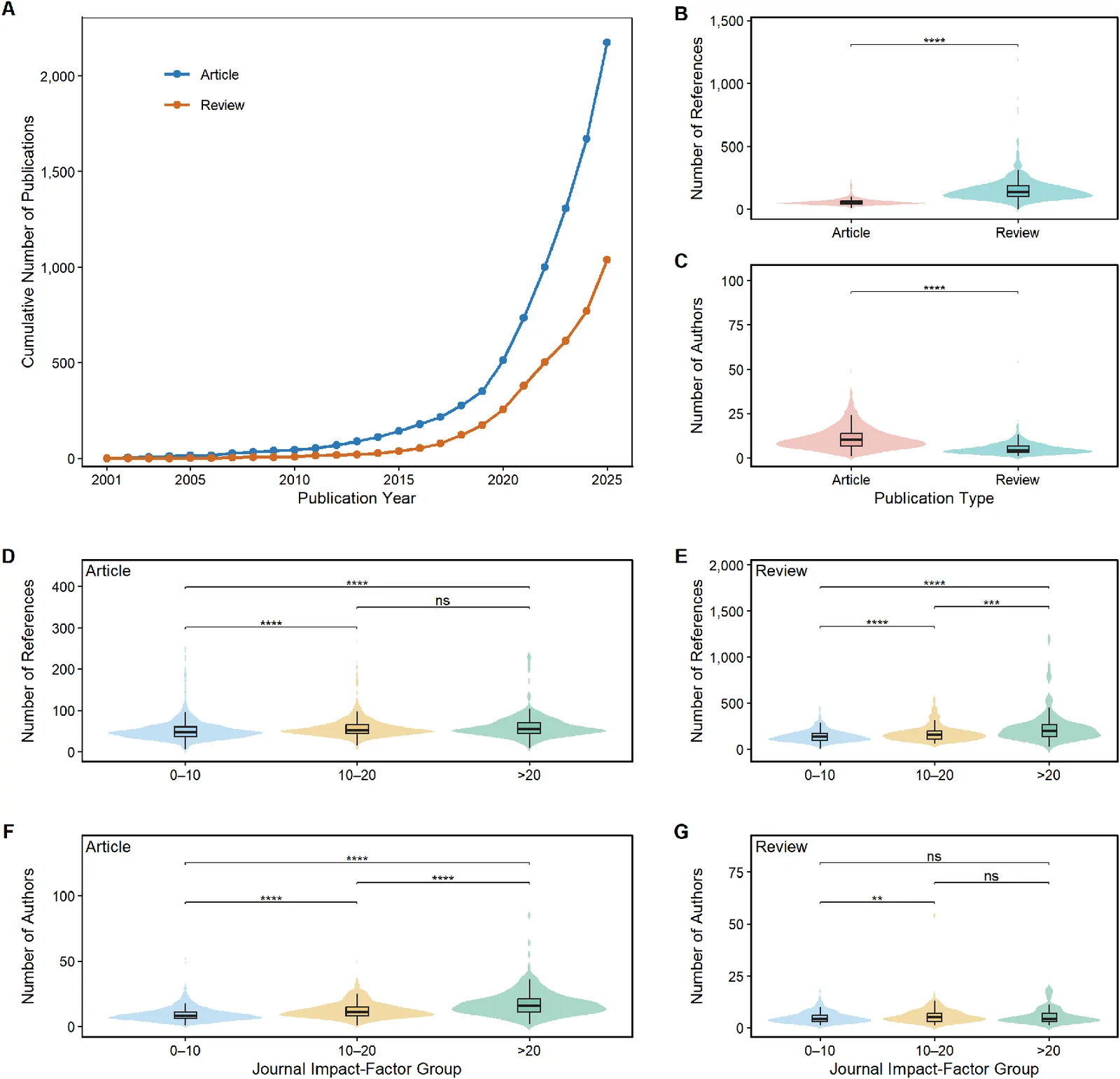
Publication type, reference use, authorship and journal-impact strata (Wo S, 2001-2025). **(A)** Cumulative number of Article (solid line) and Review (dashed line) per year. **(B, C)** Distributions of the number of references **(B)** and the number of authors **(C)** by document type. **(D–G)** Number of references **(D, E)** and number of authors **(F, G)** across 2025 journal impact factor (JIF) strata, shown separately for Articles **(D, F)** and Reviews **(E, G)**. JIF strata: 0-10 =JIF ≤ 10; 10-20 =JIF > 10 and ≤ 20; >20 = JIF >20; records without a matched JIF were excluded from these panels. In all panels, violin outlines show the kernel density of the observations and box plots show the median (center line), interquartile range (box), 1.5 × interquartile range (whiskers) and observations beyond the whiskers (points). Significance brackets show pairwise two-sided Wilcoxon rank-sum comparisons with Benjamini-Hochberg adjustment (****adjusted p < 0.0001; ***adjusted p < 0.001; **adjusted p < 0.01; ns, not significant); Brackets are shown for all pairwise comparisons, including non-significant ones. Test statistics, effect sizes and exact P values are reported in the Results and **Supplementary Table 1**.

### 3.3 Article types and study characteristics

Articles were produced by larger teams than Reviews (median 10 authors, IQR 7–14, vs 4, IQR 3–7; Wilcoxon *W* = 1,862,670.5, adjusted p < 0.001, *r*_rb_ = 0.65, large), whereas Reviews cited substantially more references (median 136, IQR 99–183.5, vs 50, IQR 40–64; Wilcoxon *W* = 148,630, adjusted *p* < 0.001, *r*_rb_ = 0.87, large; **Figures 3B,C**; **Supplementary Table 1**). These differences are consistent with the experimental and multi-platform requirements of primary tumor immunometabolism studies and the broad evidence-integration function of reviews. The Scopus dataset contained 2,324 Articles and 1,189 Reviews and independently reproduced the larger author teams in Articles (median 10 vs 4 authors; **Supplementary Figure 2**). As the reference count field is not available in Scopus export files, the comparison of reference numbers between research articles and reviews was not replicated for this database.

Within Articles, the number of references differed across journal-impact groups (Kruskal–Wallis *H* = 75.90, df = 2, *p* = 3.30 × 10^–17^, ϵ^2^ = 0.035, small). Median reference counts rose from 47 in the 0–10 group to 52 in the 10–20 group and 55 in the >20 group; the 0–10 vs 10–20 contrast (W = 302,644, adjusted p = 2.0 × 10^-12^, r_rb_ = 0.21) and the 0–10 vs >20 contrast (*W* = 141,072.5, adjusted *p* = 1.1 × 10^–10^, *r*_rb_ = 0.24) were significant with small effect sizes, whereas 10–20 vs >20 did not differ (adjusted *p* = 0.16). Team size showed a stronger gradient, increasing from a median of eight to 11 and 16 authors across the same strata (Kruskal–Wallis *H* = 352.55, df = 2, *p* = 2.78 × 10^-77^, ϵ^2^ = 0.16; all three pairwise adjusted *p* < 0.001, *r*_rb_ = 0.35, 0.32 and 0.40). Reviews also cited more references in higher-impact strata (medians 128.5, 155 and 196; Kruskal–Wallis *H* = 70.46, df = 2, *p* = 5.02 × 10^–16^, ϵ^2^ = 0.068), whereas review team size differed only modestly overall (*H* = 9.26, df = 2, *p* = 0.0098, ϵ^2^ = 0.008) and was not consistently larger in the >20 group (adjusted *p* = 0.32 for both 10–20 vs >20 and 0–10 vs >20; **Figures 3D–G**).The effect sizes are small, and these descriptive associations do not imply that impact factor causes larger teams or better science.

### 3.4 Geography and international collaboration

By corresponding-author country, China was the largest contributor in the Web of Science dataset (n=1,470; 45.7%), followed by the United States (n=727; 22.6%), Germany (n=110), Japan (n=105) and Italy (n=104; **Figures 2B**, **4A**). China and the United States together accounted for more than two-thirds of the primary corpus by corresponding-country production.

**Figure 4:**
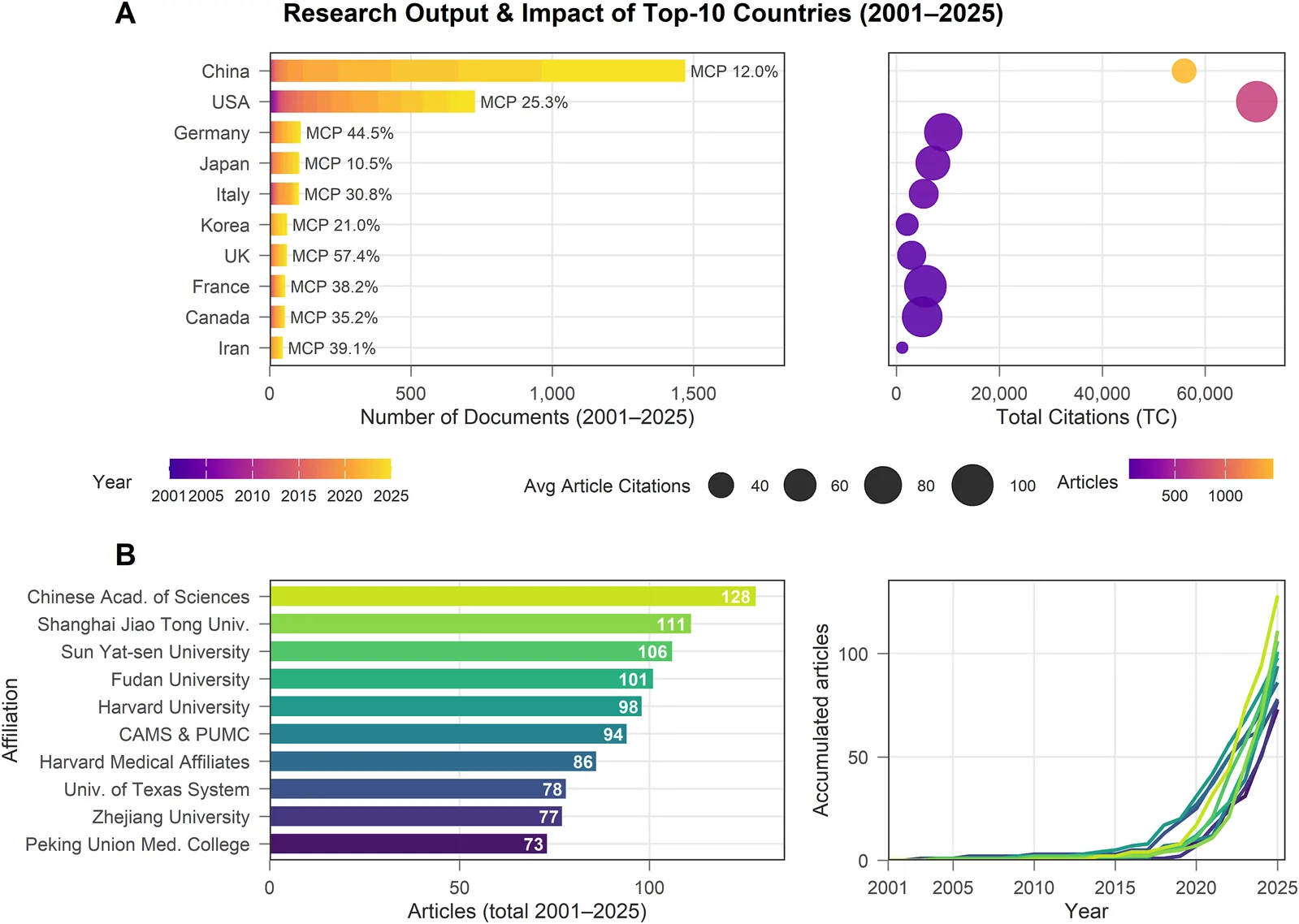
Leading countries and institutions (Wo S, 2001-2025). **(A)** Country-level publication and citation profile for the ten countries with the most publications assigned by corresponding author country. Left panel: total number of publications per country (bar length), with bars colored by publication year to show annual accumulation across 2001-2025, and the multi-country publication proportion labeled for each country as a percentage (MCP%). Right panel: total citations received (x-axis) against the same countries (y-axis), with bubble size representing the mean number of citations per publication. SCP (single-country publication) denotes a publication in which all authors are affiliated in one country; MCP (multi-country publication) denotes a publication with author affiliations in more than one country. Hong Kong SAR, Macao SAR and Taiwan Province of China are aggregated into China. **(B)** Total number of publications (upper panel) and cumulative number of publications by year (lower panel) for the ten most productive affiliations; institution counts are document-level, so each publication contributes at most one count to a given institution.

Publication volume did not parallel international collaboration or citation influence. China produced 176 multi-country publications (MCP proportion 12.0%), compared with 184 for the United States (25.3%). The United Kingdom had the highest MCP proportion among the ten leading countries (57.4%), followed by Germany (44.5%). The United States accumulated 70,049 citations and averaged 96.4 citations per article, exceeding China’s 55,911 total and 38.0 per article. France had the highest mean among the ten leading countries (101.9 citations per article) despite a much smaller publication volume. These contrasts show that numerical leadership, international connectedness and citation impact are distinct dimensions of field leadership. The Scopus analysis likewise identified China and the United States as the two largest contributors and reproduced the separation between publication volume, collaboration and citation influence (**Supplementary Figure 3**).

The Chinese Academy of Sciences was the most productive affiliation (n=128), followed by Shanghai Jiao Tong University (n=111), Sun Yat-sen University (n=106), Fudan University (n=101) and Harvard University (n=98; **Figure 4B**). Institution-level VO Sviewer mapping showed dense links among major Chinese universities and a distinct but connected North American grouping (**Supplementary Figure 7A**). However, affiliation aggregation is sensitive to institutional naming, umbrella systems and multi-affiliation counting.

### 3.5 Author collaboration networks

The author co-authorship map contained 27 threshold-eligible authors but only 23 links and 14 clusters, indicating that productivity was distributed across multiple teams rather than one densely integrated global network (**Supplementary Figure 7B**). A connected group included Ho, Rathmell, Pedro Romero and Delgoffe, whereas several productive authors appeared weakly connected or isolated at the selected threshold.

### 3.6 Journal distribution, impact factors and JCR quartiles

Frontiers in Immunology published the largest number of records (n=282), comprising 112 Articles and 170 Reviews (**Figure 5A**). It was followed by Journal for ImmunoTherapy of Cancer (n=99), Frontiers in Oncology (n=87), Cancers (n=79) and International Journal of Molecular Sciences (n=59). Six of the ten leading journals were classified as JCR Q1 in the matched 2025 data. Nature Communications (n=52; JIF 18.1) and Cancer Research (n=42; JIF 22.6) combined lower publication volume with high journal-level citation visibility.

**Figure 5:**
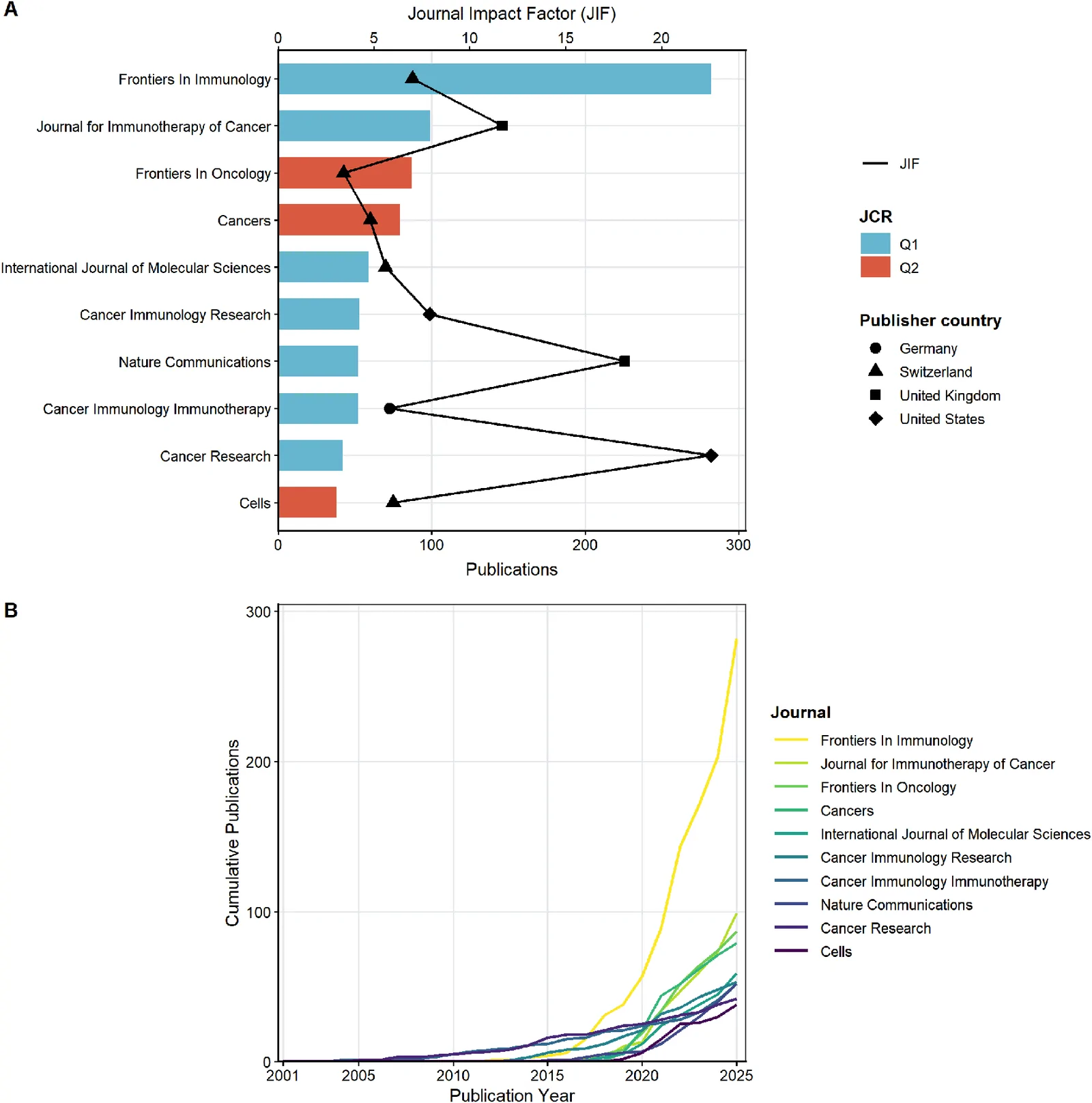
Leading journals and temporal publication dynamics (Wo S, 2001-2025). **(A)** The ten most productive journals. Each journal has a bar showing its total number of publications (lower x-axis), split into Articles and Reviews, a point showing its 2025 journal impact factor (JIF; upper secondary x-axis) with points joined by a line to aid reading, the bar fill indicating the 2025 Journal Citation Reports (JCR) quartile (blue = Q1, red = Q2) and point shape indicating the publisher country. **(B)** Cumulative number of publications per year for each of the ten journals (one line per journal). JIF and quartiles are journal-level attributes for the 2025 JCR edition and are not measures of the quality of individual studies.

The annual journal curves indicate that the distribution was not static (**Figure 5B**). Frontiers in Immunology expanded most rapidly and became a principal synthesis and dissemination venue, while more selective immunology, oncology and general-science journals contributed fewer but often highly cited mechanistic studies. Scopus reproduced the leading positions of Frontiers in Immunology and Journal for ImmunoTherapy of Cancer and showed a closely aligned top-journal distribution (**Supplementary Figure 4**).

### 3.7 Author productivity, citation impact, and temporal activity

Ping-Chih Ho was the most productive normalized author in the curated table, with 29 publications, a dataset H-index of 25 (the Hirsh index recalculated only within the publications included in this corpus, not the author’s overall H-index) and 6,419 recalculated citations from 2015-2025 (**Figure 6**). Yi Zhang contributed 23 deduplicated publications (dataset H-index 18), and Greg M. Delgoffe contributed 22 (dataset H-index 19; 4,447 citations). Jeffrey C. Rathmell combined 18 publications with 4,221 citations. Several Chinese authors entered the top group later, particularly during 2020-2025, reflecting the recent expansion of the national research base. The Scopus ranking was broadly concordant: Ping-Chih Ho remained the most productive author (n=30), followed by Yi Zhang (n=25), with Greg M. Delgoffe and Jeffrey C. Rathmell also retained among the leading contributors (**Supplementary Figure 5**).

**Figure 6:**
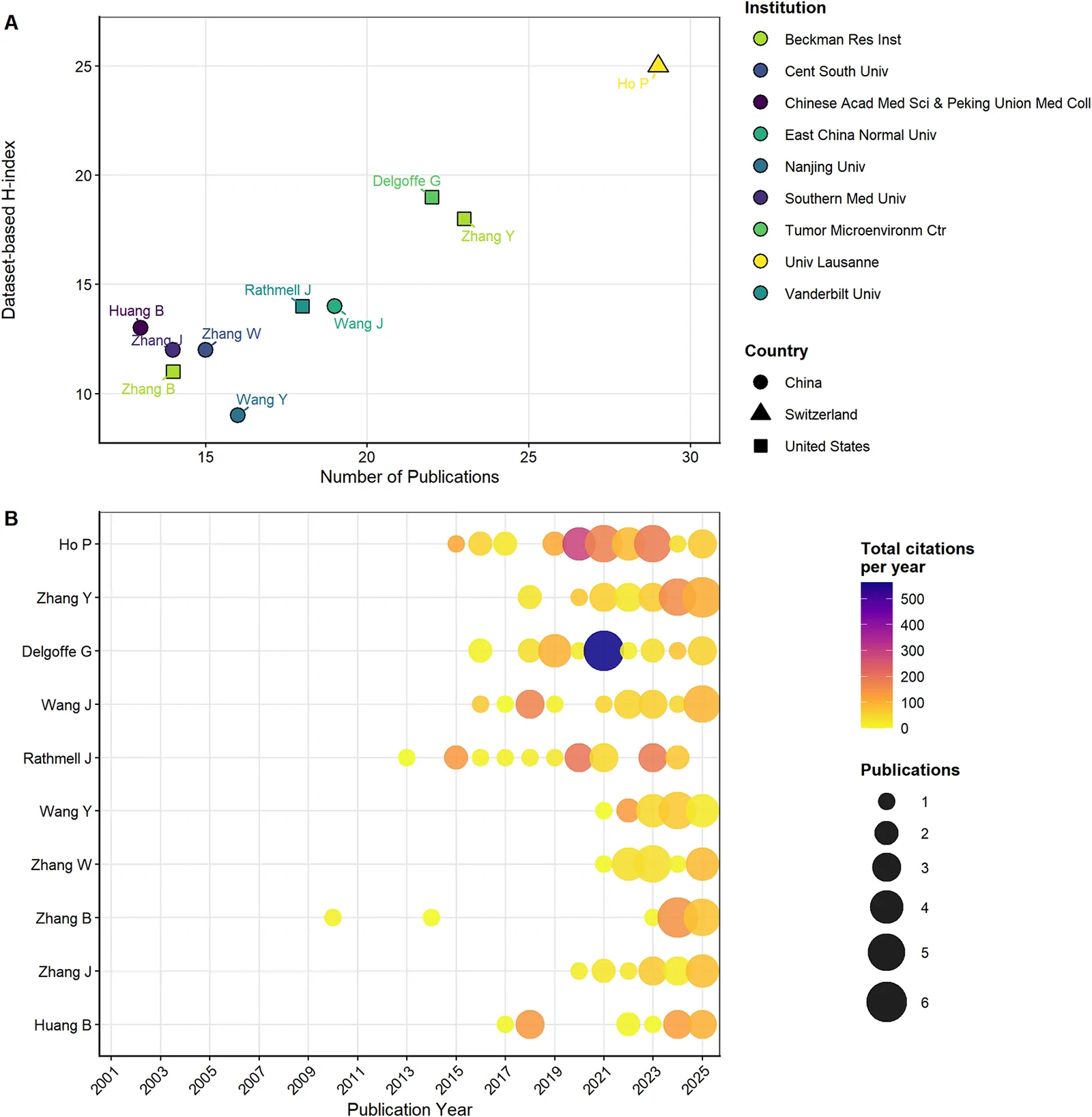
Productivity and temporal activity of leading authors (Wo S, 2001-2025). **(A)** Number of publications (x-axis) against the dataset H-index (y-axis) for the ten most productive normalized authors; each point is one author, labeled with the author’s name, colored by the corresponding author’s institution and shaped by country. The dataset H-index is the Hirsch index recalculated only within the publications included in this corpus, not the author’s overall H-index. **(B)** Annual author activity for the same authors (one row per author, y-axis); bubble size represents the number of publications by that author in that year and bubble color represents the number of citations received in that year, from light (fewer) to dark (more). Bubble size and color are the only encoding in panel **(B)** Author names were normalized and variants merged before counting.

### 3.8 Reference citation bursts and trend evolution

More recent highly normalized or burst-active studies focused on mitochondrial fitness, persistent-antigen exposure, T-cell exhaustion, lactate-supported regulatory T cells and memory T-cell differentiation (12, 13, 21, 22). This citation sequence supports a transition in the field’s intellectual base: initial work established that metabolic conditions control antitumor immunity; subsequent studies identified intervention points that could be incorporated into checkpoint therapy or engineered cellular products (**Supplementary Figure 7C**).

### 3.9 Highly cited publications

The most globally cited record was Chang et al.’s 2015 Cell study, “Metabolic Competition in the Tumor Microenvironment Is a Driver of Cancer Progression” (2,727 global and 447 local citations) (**Supplementary Table 2**). Ho et al.’s parallel Cell study on phosphoenolpyruvate as a metabolic checkpoint ranked second by local citations (279). Other highly influential work linked PD-1 signaling to altered T-cell metabolism (7), hypoxia to PD-L1 regulation (8), costimulatory signaling to CAR-T-cell metabolic memory (9), and glutamine blockade to divergent tumor and immune-cell programs (11).

### 3.10 Keyword co-occurrence network and thematic clusters

The VO Sviewer network organized 64 threshold-eligible author keywords into six interconnected clusters (**Supplementary Figure 7D**). At a higher conceptual level, these clusters linked: (i) T-cell activation, memory and exhaustion; (ii) macrophage and myeloid-cell polarization; (iii) hypoxia, lactate and adenosine-rich microenvironments; (iv) immune checkpoint blockade; (v) CAR-T and adoptive-cell strategies; and (vi) cancer-specific biomarker and prognosis applications. Tumor microenvironment, immunotherapy, immunometabolism and metabolic reprogramming served as prominent bridges. In this network, metabolism and immunotherapy terms co-occurred rather than forming separate clusters.

### 3.11 Citation burst analysis of keywords

After synonym normalization, the most frequent author keyword was immunotherapy (n=768), followed by tumor microenvironment (n=469), metabolism (n=198), T cells (n=187), cancer (n=168), immunometabolism (n=155), hypoxia (n=149), cancer immunotherapy (n=141), metabolic reprogramming (n=133), immune checkpoint inhibitors (n=121) and CAR-T cells (n=101; **Figure 7A**).

**Figure 7:**
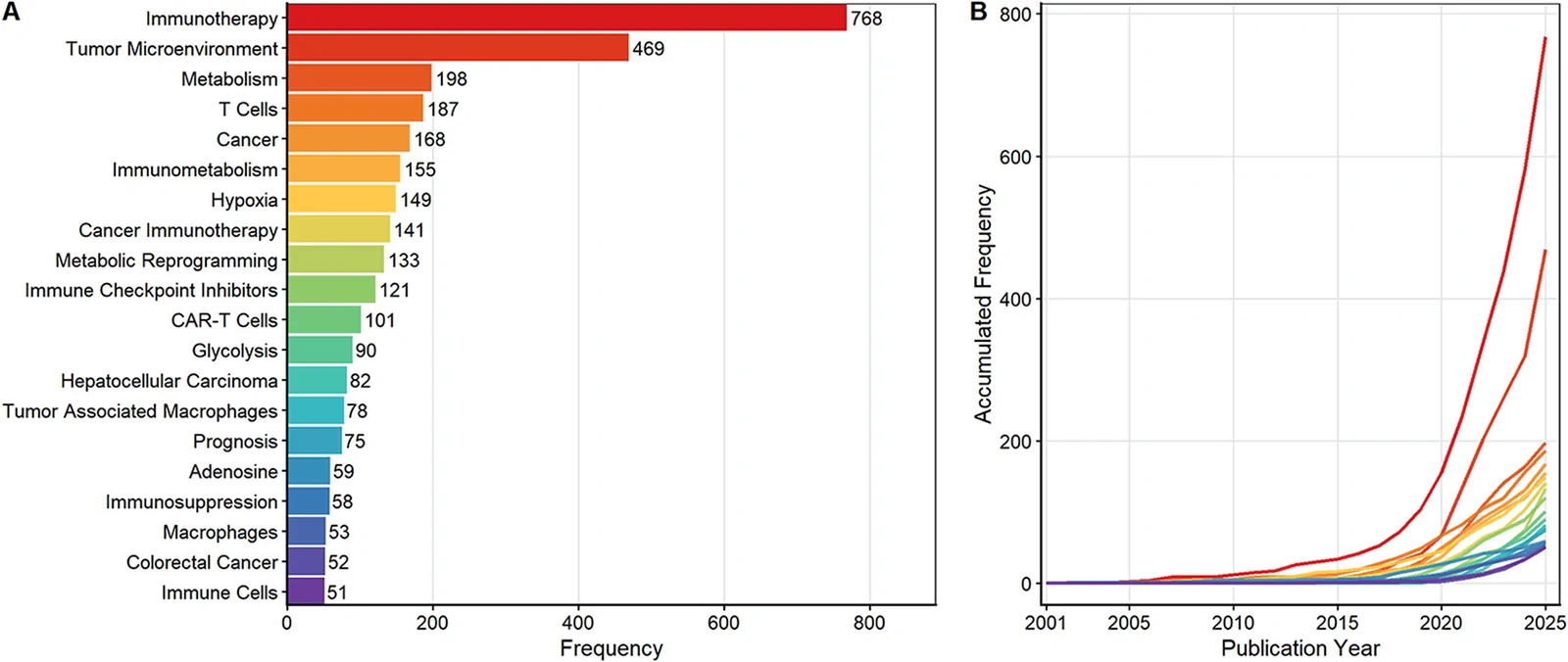
Author-keyword frequency and temporal evolution (Wo S, 2001-2025). **(A)** The 20 most frequent author keywords after synonym normalization, with the number of records containing each keyword on the x-axis (frequency); a documented synonym dictionary was applied so that variants (for example CAR-T/CAR-T cells) were merged before counting. A single color scale is shared by both panels: each keyword is assigned one color according to tis frequency rank, from red (rank 1, highest frequency) though orange, yellow, green and cyan to blue and purple (rank 20, lowest frequency). **(B)** Cumulative frequency of records per year containing each plotted keyword, from 2001 to 2025 (one line per keyword). Keyword assignment is based on the author keywords indexed in the source; only records containing author keywords contribute to these counts.

The cumulative trajectories accelerated after 2020 (**Figure 7B**). Scopus independently identified immunotherapy, tumor microenvironment, metabolism, immunometabolism and metabolic reprogramming among the leading keywords and reproduced their recent growth (**Supplementary Figure 6**). Annual use of immunotherapy increased from 51 in 2020 to 187 in 2025, and tumor microenvironment increased from 26 to 150. Metabolic reprogramming rose from three annual occurrences in 2020 to 58 in 2025. Immune checkpoint inhibitors increased from 12 to 32, CAR-T cells from seven to 28, and glycolysis from five to 25. In contrast, adenosine increased more gradually, consistent with an established pathway that remains relevant but is no longer the sole framing of the field.

## 4 Discussion

This bibliometric analysis identifies a three-phase evolutionary structure in research on the tumor immunometabolism–cancer immunotherapy nexus. The field first concentrated on metabolic constraints and immune suppression, then moved toward actionable metabolic checkpoints, and most recently expanded into cellular fitness, treatment resistance and metabolism-informed engineering. The transition is supported by concordant post-2020 growth in two databases, broadly aligned country, journal, author and keyword rankings across Web of Science and Scopus, a citation sequence extending from metabolic competition to mitochondrial and cellular-therapy fitness, and the accelerating use of metabolic reprogramming, immune checkpoint inhibitor and CAR-T terminology. Thus, the principal change is conceptual as well as numerical: metabolism is increasingly treated as a measurable and engineerable component of immunotherapy design.

The evidence supports a three-stage interpretation, although the boundaries are approximate and reflect citation uptake rather than strict discovery dates.

Early work established the pathways through which the tumor microenvironment limits immune activity. Adenosine signaling, oxygen deprivation, nutrient availability and myeloid suppression were prominent. Noman et al. linked HIF-1α to PD-L1 regulation under hypoxia (8), illustrating how a metabolic condition could directly intersect with an immune checkpoint. Recent work has further shown that hypoxic programs reshape effector-cell states and can accompany checkpoint resistance (23–26). During this stage, adoptive immunotherapy and *in-vivo* immune function appeared as early burst terms, but the metabolic literature was still primarily explanatory rather than engineering-oriented.

The second stage was defined by influential studies showing that immune and tumor cells compete for metabolites and that T-cell metabolism contains actionable checkpoints. Chang et al. demonstrated the consequences of glucose competition in the tumor microenvironment (5), while Ho et al. identified phosphoenolpyruvate as a checkpoint of antitumor T-cell responses (6). PD-1-associated metabolic reprogramming (7) and CAR costimulation-dependent metabolic fate (9) connected pathway biology to immunotherapy design. Glutamine blockade subsequently showed that a metabolic intervention could produce divergent effects in tumor and immune cells, providing a conceptual route to overcome immune evasion (11).

This stage reframed metabolism from a background property to a therapeutic design variable. Nevertheless, most evidence remained preclinical. A metabolic target that improves T-cell function in a controlled model may have different effects across tumor cells, stromal populations and systemic tissues. That tension remains central to translation.

The third stage emphasized persistent-antigen exposure, mitochondrial dysfunction, exhaustion, regulatory T-cell adaptation and cellular-product fitness. Hypoxia-associated mitochondrial stress can accelerate dysfunctional T-cell states (12). Lactate was shown not only as a waste product but also as a substrate supporting tumor-infiltrating regulatory T cells and PD-1 expression (21, 22). The mitochondrial pyruvate carrier further connected metabolic routing to memory differentiation and antitumor function (13).

These studies align with the bibliometric rise of metabolic reprogramming, immune checkpoint inhibitors and CAR-T cells. The translational agenda now includes at least four strategies: selecting patients with metabolically informative biomarkers; combining checkpoint blockade with metabolic intervention; engineering cellular therapies for nutrient-poor or hypoxic tumors; and monitoring systemic toxicity when targeting pathways shared by malignant and normal immune cells.

Recent evidence supports the bibliometric convergence of metabolic stress, immune-cell state and therapeutic design. T-cell and natural-killer-cell fitness are shaped by lipid handling, amino-acid availability and regulatory-cell adaptation, while metabolite sensing can alter differentiation, persistence and immune escape (3, 10, 27–37). These studies justify treating immune-cell metabolism as a collection of cell-state-specific programs rather than a single pathway.

Hypoxia, lactate and adenosine remain central because they connect local physicochemical conditions to immune dysfunction and treatment response. The effects are context dependent: hypoxia can alter surface and effector programs, lactate can suppress or support distinct immune populations, and adenosine-receptor blockade can restore cytotoxicity in metabolically perturbed tumors (4, 23–26, 38–40). This heterogeneity argues against assuming that one metabolic intervention will act uniformly across all tumor and immune compartments.

Amino-acid and stromal metabolism broaden the ecosystem view. Competition for glutamine, arginine and branched-chain amino acids intersects with B-cell, macrophage, fibroblast and endothelial programs, and these interactions can influence T-cell recruitment and function (41–51). The bibliometric rise of metabolic reprogramming therefore reflects a shift from isolated pathway studies toward multicellular nutrient exchange.

Checkpoint resistance is also being reframed metabolically. Recent work links metabolic checkpoint modulation, TIGIT-associated T-cell programs, tumor-derived metabolic gatekeepers and CD8 T-cell rewiring to strategies intended to restore antitumor immunity (16, 52–59). These findings support the prominence of immune checkpoint inhibitor terminology, but most remain mechanistic or preclinical and should not be interpreted as proof of patient benefit.

CAR-T research provides the clearest engineering-oriented branch. Studies and reviews now address mitochondrial state, kynurenine degradation, endothelial serine metabolism, branched-chain amino-acid routing and even the effect of CAR extracellular domains on basal T-cell metabolism (60–70). Together, they motivate manufacturing and product-release strategies that connect ex-vivo metabolic fitness with persistence in nutrient-poor solid tumors.

Finally, spatial metabolomics, patient-derived cellular analyses and multi-omics are beginning to connect pathway mechanisms with clinically anchored tissue states (15, 71, 72). Their translational value will depend on prospective validation, standardized sampling and explicit separation of predictive biomarkers from causal intervention targets.

China’s rapid growth has made it the largest publication contributor, supported by several high-output institutions. The United States retained greater citation impact and a higher international-collaboration proportion. European countries such as the United Kingdom and Germany showed particularly high MCP proportions despite smaller absolute output. These differences suggest complementary strengths: large discovery pipelines in China, highly cited mechanistic and translational centers in the United States, and cross-border connectivity in parts of Europe.

The author network remained fragmented at the applied threshold. Interpreted cautiously, this topological collaboration pattern indicates that translational immunometabolism research is dispersed across independent research teams with limited co-authorship overlap. This structural separation may partially explain the existing disconnect between basic laboratory pathway investigations and clinically anchored datasets, implying that multinational collaborative consortia, unified protocols, and standardized prospective metabolic outcome definitions could help bridge this divide in future studies.

Beyond the thematic synthesis above, the record also shows where the published coverage is thin or unbalanced. The four points below identify the gaps that follow most directly from the evidence reported in this study.

First, the field needs measurements that are robust across platforms. Metabolic gene signatures are not equivalent to flux, and bulk-tissue abundance can obscure cell-specific nutrient use. Prospective studies should integrate spatial metabolite mapping, isotope tracing where feasible, single-cell profiling and functional immune assays (71, 72).

Second, interventions should be evaluated as ecosystem perturbations. Blocking glycolysis, glutamine metabolism, fatty-acid oxidation, lactate transport or adenosine signaling can affect tumor cells, effector T cells, regulatory T cells, macrophages and normal tissues in different directions. Preclinical designs should therefore measure competing cell populations rather than report a single immune endpoint (41, 43, 49).

Third, cellular therapies require manufacturing-to-tumor continuity. Metabolic fitness established during CAR-T-cell manufacturing may not persist after exposure to hypoxia, low glucose, acidosis and chronic antigen. Release criteria that include mitochondrial reserve, substrate flexibility and memory-state markers could be tested against clinical persistence and response (60, 65, 70).

Fourth, clinical studies should distinguish biomarker utility from therapeutic causality. A metabolite or metabolic program may predict response without being a safe intervention target. Trials should prespecify whether the objective is patient stratification, pharmacodynamic monitoring or direct metabolic manipulation.

The critical reading of this record is less favorable than its growth curve. The literature is concentrated in a small number of countries and institutions while the author network remains fragmented, so the available evidence is produced by a limited set of groups working on largely separate problems. Within that output, mechanism and engineering studies are predominantly preclinical or early translational, and proof-of-concept mechanism is over-represented relative to safety and toxicity reporting for targets shared between malignant and immune cells. Measurement practice is a further weakness, because transcriptional signatures and bulk-tissue abundance are used far more often than flux measurement or spatial metabolite mapping, and the two are not equivalent. Positive mechanism-oriented findings also dominate, while neutral and negative results are rarely visible, which makes the apparent internal consistency of the field difficult to evaluate. Keyword frequency and citation-burst trends show rapid adoption of terms such as metabolic reprogramming, immune checkpoint inhibitors and CAR-T cells, but the same record provides little information on trial success, regulatory readiness or patient benefit, and bibliometric frequency should not be read as evidence of clinical effectiveness.

This study has several limitations. First, bibliometric coverage depends on database indexing and the search vocabulary. The strategy intentionally required cancer, immune-metabolic and immunotherapy concepts, which increases relevance to translation but may exclude foundational metabolic studies that did not use immunotherapy language. Second, only English-language journal Articles and Reviews were included; conference abstracts, books and non-English work were excluded. Third, the 2025 output is complete only to the extent that records had been indexed by the July 2026 search date, and database updates may change counts. Fourth, because counts are by corresponding-author country, countries that participate mainly as non-corresponding collaborators are under-represented relative to a full-affiliation count. Fifth, citation counts favor older publications and can be influenced by journal visibility and field size. Journal impact factor is a journal-level descriptor, not a study-quality measure. Sixth, country, institution and author analyses are sensitive to name disambiguation, multi-affiliation counting and institutional umbrella structures. A repeated-author record for Yi Zhang required deduplication, and other common names may retain residual ambiguity. Seventh, keyword normalization and VO Sviewer thresholds necessarily exclude some low-frequency or very recent terms. The supplied Cite Space cluster output had silhouette values of zero; we therefore did not use its cluster labels as confirmatory thematic evidence. Finally, bibliometric movement toward checkpoint inhibitors, CAR-T cells and metabolic intervention does not quantify clinical trial success, regulatory readiness, safety or patient benefit. The phrase “clinical translation” in this manuscript denotes a shift in research orientation, not demonstrated clinical effectiveness. Traditional clinical risk-of-bias instruments were not applied because the unit of analysis was the bibliographic record and no intervention effects were synthesized; consequently, the review characterizes the structure and direction of the literature but cannot establish the validity of individual clinical claims.

## 5 Conclusion

Worldwide research on the tumor immunometabolism–cancer immunotherapy nexus expanded rapidly after 2020 and developed from an early emphasis on metabolic immune suppression into a broader program of checkpoint modulation, cellular fitness and engineered therapy. China leads publication volume, the United States retains greater citation influence, and international collaboration remains uneven. The next stage should connect cell-specific metabolic measurements with prospective clinical endpoints and distinguish predictive biomarkers from safe causal targets. The bibliometric record supports a clear translation-oriented shift in the literature; clinical benefit, safety and readiness nevertheless require appropriately designed experimental and clinical studies.

## Data Availability

The datasets generated for this study are deposited in ScienceDB (https://www.scidb.cn/) and are publicly available at https://doi.org/10.57760/sciencedb.013cf. Requests to access these datasets should be directed to the corresponding author.

## Supplementary material

Supplementary Methods Search Strategy.

Supplementary Table 1 All statistical comparisons reported in the manuscript.

Supplementary Table 2 Top 10 globally and locally cited documents.

Supplementary Figure 1 Scopus annual publication growth and geographic distribution.

Supplementary Figure 2 Scopus document types, authorship characteristics and journal-impact strata.

Supplementary Figure 3 Scopus country and institutional contributions.

Supplementary Figure 4 Scopus journal distribution and publication dynamics.

Supplementary Figure 5 Scopus author productivity and temporal activity.

Supplementary Figure 6 Scopus keyword frequency and temporal trends.

Supplementary Figure 7 Web of Science collaboration networks, reference citation bursts, and author-keyword co-occurrence network.
