# Supplementary material for: Worldwide research trends on the tumor immunometabolism–cancer immunotherapy nexus: a systematic bibliometric review
Source: Front Oncol. 2026 Oct 19;16:1962391. doi: 10.3389/fonc.2026.1962391 (PMC13724477; doi:10.3389/fonc.2026.1962391)
Supplement: Supplementary file 1 [file DataSheet1.docx]

Supplementary Methods: Complete Database Search Strategies

Search date: 26 July 2026.

Eligible publication years: 2001–2025.

Searches were designed around malignancy, immune-cell metabolism and cancer immunotherapy.

Web of Science Core Collection and Scopus were analyzed as separate corpora.

**Web of Science Core Collection：**

TS=((tumor* OR tumour* OR cancer* OR neoplasm* OR carcinoma* OR malignan* OR leukemia* OR leukaemia* OR lymphoma* OR myeloma*) AND ( immunometabol* OR "immune metabolism" OR ( ( metabol* OR glycoly* OR "pentose phosphate" OR "oxidative phosphorylation" OR lipid* OR "fatty acid*" OR glutamin* OR arginin* OR tryptophan* OR kynurenin* OR lactat* OR adenosine OR hypoxi* OR redox ) NEAR/5 ( "immune cell*" OR "T cell*" OR "CD8* T cell*" OR "CD4* T cell*" OR lymphocyte* OR "regulatory T cell*" OR Treg* OR macrophage* OR "myeloid-derived suppressor cell*" OR MDSC* OR "dendritic cell*" OR "natural killer cell*" OR "NK cell*" )) OR ("metabolic competition" NEAR/5 immun*) OR ("metabolic checkpoint*" NEAR/5 immun*)) AND ( immunotherap* OR "immune checkpoint blockade" OR "immune checkpoint inhibitor*" OR "checkpoint immunotherap*" OR "CAR-T" OR "CAR T cell*" OR "chimeric antigen receptor T cell*" OR "TCR-T" OR "TCR T cell*" OR "adoptive cell therap*" OR ((PD-1 OR PD-L1 OR CTLA-4) NEAR/5 (blockad* OR inhibit* OR therap* OR treat* OR resist* OR response*))))

**Scopus：**

TITLE-ABS-KEY ( ( tumor* OR tumour* OR cancer* OR neoplasm* OR carcinoma* OR malignan* OR leukemia* OR leukaemia* OR lymphoma* OR myeloma* ) AND ( immunometabol* OR "immune metabolism" OR ( ( metabol* OR glycoly* OR "pentose phosphate" OR "oxidative phosphorylation" OR lipid* OR "fatty acid*" OR glutamin* OR arginin* OR tryptophan* OR kynurenin* OR lactat* OR adenosine OR hypoxi* OR redox ) W/5 ( "immune cell*" OR "T cell*" OR "CD8* T cell*" OR "CD4* T cell*" OR lymphocyte* OR "regulatory T cell*" OR Treg* OR macrophage* OR "myeloid-derived suppressor cell*" OR MDSC* OR "dendritic cell*" OR "natural killer cell*" OR "NK cell*" ) ) OR ( "metabolic competition" W/5 immun* ) OR ( "metabolic checkpoint*" W/5 immun* ) ) AND ( immunotherap* OR "immune checkpoint blockade" OR "immune checkpoint inhibitor*" OR "checkpoint immunotherap*" OR "CAR-T" OR "CAR T cell*" OR "chimeric antigen receptor T cell*" OR "TCR-T" OR "TCR T cell*" OR "adoptive cell therap*" OR ( ( PD-1 OR PD-L1 OR CTLA-4 ) W/5 ( blockad* OR inhibit* OR therap* OR treat* OR resist* OR response* ) ) ) ) AND PUBYEAR > 2000 AND PUBYEAR < 2026 AND ( DOCTYPE ( ar ) OR DOCTYPE ( re ) ) AND PUBYEAR > 2000 AND PUBYEAR < 2026

**Eligibility limits applied after export**

**Web of Science**: English-language Articles or Reviews; 3,386 records identified, 172 excluded (164 other document types and 8 non-English), 3,214 retained. **Scopus**: English-language journal records; 3,602 records identified, 89 excluded (79 non-English and 10 non-journal source types), 3,513 retained. No unpublished material, submitted manuscripts or personal communications were eligible. The two databases were not pooled, so cross-database deduplication was not used to calculate a combined sample.

Note: The Scopus search string already contains document-type and publication-year limits. The reported post-export eligibility counts reflect the final exported metadata and the prespecified harmonization rules used for the parallel database analyses.
