# Supplementary material for: Worldwide research trends on the tumor immunometabolism–cancer immunotherapy nexus: a systematic bibliometric review
Source: Front Oncol. 2026 Oct 19;16:1962391. doi: 10.3389/fonc.2026.1962391 (PMC13724477; doi:10.3389/fonc.2026.1962391)
Supplement: Supplementary file 2 [file Table1.docx]

**Supplementary Table 1.** All statistical comparisons reported in the manuscript

| **Outcome** | **Comparison** | **n (group 1 vs group 2)** | **Test** | **Test statistic** | **Effect size** | **Exact P** | **BH-adjusted P** |
| --- | --- | --- | --- | --- | --- | --- | --- |
| Number of references | Articles (0–10 vs 10–20 vs >20) | 2160 | Kruskal–Wallis | *H* = 75.90 (df = 2) | ε² = 0.035 (negligible) | 3.301 × 10⁻¹⁷ | — |
| Number of references | Reviews (0–10 vs 10–20 vs >20) | 1030 | Kruskal–Wallis | *H* = 70.46 (df = 2) | ε² = 0.068 (negligible) | 5.022 × 10⁻¹⁶ | — |
| Number of authors | Articles (0–10 vs 10–20 vs >20) | 2160 | Kruskal–Wallis | *H* = 352.55 (df = 2) | ε² = 0.163 (small) | 2.783 × 10⁻⁷⁷ | — |
| Number of authors | Reviews (0–10 vs 10–20 vs >20) | 1030 | Kruskal–Wallis | *H* = 9.26 (df = 2) | ε² = 0.009 (negligible) | 9.774 × 10⁻³ | — |
| Number of references | Articles vs Reviews, Article vs Review | 2175 vs 1039 | Wilcoxon rank-sum | *W* = 148,630 | *r*_rb_ = -0.868 (large) | < 2.2 × 10⁻¹⁶ | < 2.2 × 10⁻¹⁶ |
| Number of authors | Articles vs Reviews, Article vs Review | 2175 vs 1039 | Wilcoxon rank-sum | *W* = 1,862,670.5 | *r*_rb_ = +0.649 (large) | < 2.2 × 10⁻¹⁶ | < 2.2 × 10⁻¹⁶ |
| Number of references | Articles, 0–10 vs 10–20 | 1259 vs 605 | Wilcoxon rank-sum | *W* = 302,644 | *r*_rb_ = -0.205 (small) | 6.526 × 10⁻¹³ | 1.958 × 10⁻¹² |
| Number of references | Articles, 0–10 vs >20 | 1259 vs 296 | Wilcoxon rank-sum | *W* = 141,072.5 | *r*_rb_ = -0.243 (small) | 7.419 × 10⁻¹¹ | 1.113 × 10⁻¹⁰ |
| Number of references | Articles, 10–20 vs >20 | 605 vs 296 | Wilcoxon rank-sum | *W* = 84,404.5 | *r*_rb_ = -0.057 (negligible) | 0.1615 | 0.1615 |
| Number of references | Reviews, 0–10 vs 10–20 | 754 vs 175 | Wilcoxon rank-sum | *W* = 51,100 | *r*_rb_ = -0.225 (small) | 3.294 × 10⁻⁶ | 4.942 × 10⁻⁶ |
| Number of references | Reviews, 0–10 vs >20 | 754 vs 101 | Wilcoxon rank-sum | *W* = 20,513 | *r*_rb_ = -0.461 (medium) | 4.853 × 10⁻¹⁴ | 1.456 × 10⁻¹³ |
| Number of references | Reviews, 10–20 vs >20 | 175 vs 101 | Wilcoxon rank-sum | *W* = 6,423.5 | *r*_rb_ = -0.273 (small) | 0.0001577 | 0.0001577 |
| Number of authors | Articles, 0–10 vs 10–20 | 1259 vs 605 | Wilcoxon rank-sum | *W* = 247,315 | *r*_rb_ = -0.351 (medium) | 8.962 × 10⁻³⁵ | 1.344 × 10⁻³⁴ |
| Number of authors | Articles, 0–10 vs >20 | 1259 vs 296 | Wilcoxon rank-sum | *W* = 73,003 | *r*_rb_ = -0.608 (large) | 5.523 × 10⁻⁶⁰ | 1.657 × 10⁻⁵⁹ |
| Number of authors | Articles, 10–20 vs >20 | 605 vs 296 | Wilcoxon rank-sum | *W* = 57,786.5 | *r*_rb_ = -0.355 (medium) | 4.456 × 10⁻¹⁸ | 4.456 × 10⁻¹⁸ |
| Number of authors | Reviews, 0–10 vs 10–20 | 754 vs 175 | Wilcoxon rank-sum | *W* = 56,556.5 | *r*_rb_ = -0.143 (small) | 0.002978 | 0.008935 |
| Number of authors | Reviews, 0–10 vs >20 | 754 vs 101 | Wilcoxon rank-sum | *W* = 35,502.5 | *r*_rb_ = -0.068 (negligible) | 0.2652 | 0.319 |
| Number of authors | Reviews, 10–20 vs >20 | 175 vs 101 | Wilcoxon rank-sum | *W* = 9,469.5 | *r*_rb_ = +0.072 (negligible) | 0.319 | 0.319 |

Dataset: Web of Science Core Collection, final eligible sample (*N* = 3,214; 2,175 Articles and 1,039 Reviews). JIF strata: 0–10 = JIF ≤ 10; 10–20 = JIF > 10 and ≤ 20; >20 = JIF > 20. All comparisons are two-sided. *W* is the Wilcoxon rank-sum statistic and *H* the Kruskal–Wallis statistic, both exactly as reported by the analysis script. *r*_rb_ = 2*W*/(*n*₁*n*₂) − 1 and ε² = *H*/((*n*² − 1)/(*n* + 1)); the sign of *r*_rb_ follows the first-named group. Benchmarks for |*r*_rb_| and ε²: 0.10, 0.30 and 0.50 indicate small, medium and large effects. Multiple comparisons were adjusted using the Benjamini–Hochberg (BH) method at a false-discovery rate of 0.05. Family 1 comprised the two document-type comparisons (Article vs Review) for the number of references and the number of authors; Family 2 comprised the 12 pairwise comparisons among JIF strata, tested separately for each document type and each available outcome. The two families were corrected independently, no comparison appeared in more than one family, and the Kruskal–Wallis omnibus tests were not included in either family. Two comparisons reported *P* = 0 in the analysis output because the normal approximation fell below the smallest representable double-precision value; these are given as *P* < 2.2 × 10⁻¹⁶. Effect sizes were computed from the reported test statistics and group sizes. Panel numbers refer to Figure 3.
