# Supplementary material for: Worldwide research trends on the tumor immunometabolism–cancer immunotherapy nexus: a systematic bibliometric review
Source: Front Oncol. 2026 Oct 19;16:1962391. doi: 10.3389/fonc.2026.1962391 (PMC13724477; doi:10.3389/fonc.2026.1962391)
Supplement: Supplementary file 3 [file Table2.docx]

**Supplementary Table 2** The top 10 globally and locally cited documents

| **Paper** | **DOI** | **Year** | **Local Citations** | **Global Citations** | **LC/GC Ratio (%)** | **Normalized Local Citations** | **Normalized Global Citations** |
| --- | --- | --- | --- | --- | --- | --- | --- |
| CHANG CH, 2015, CELL | 10.1016/j.cell.2015.08.016 | 2015 | 447 | 2727 | 16.39164 | 13.36228 | 11.98556 |
| HO PC, 2015, CELL | 10.1016/j.cell.2015.08.012 | 2015 | 278 | 1233 | 22.54663 | 8.31032 | 5.419213 |
| PATSOUKIS N, 2015, NAT COMMUN | 10.1038/ncomms7692 | 2015 | 227 | 1014 | 22.38659 | 6.785765 | 4.456676 |
| LEONE RD, 2019, SCIENCE | 10.1126/science.aav2588 | 2019 | 200 | 982 | 20.3666 | 16.26545 | 6.844923 |
| WATSON MJ, 2021, NATURE | 10.1038/s41586-020-03045-2 | 2021 | 173 | 1082 | 15.98891 | 26.71095 | 14.52462 |
| SCHARPING NE, 2021, NAT IMMUNOL | 10.1038/s41590-020-00834-9 | 2021 | 145 | 752 | 19.28191 | 22.38779 | 10.09475 |
| NOMAN MZ, 2014, J EXP MED | 10.1084/jem.20131916 | 2014 | 143 | 1866 | 7.663451 | 12.19706 | 12.14953 |
| ZHANG Y, 2017, CANCER CELL | 10.1016/j.ccell.2017.08.004 | 2017 | 143 | 551 | 25.95281 | 8.444337 | 4.341385 |
| YANG W, 2016, NATURE | 10.1038/nature17412 | 2016 | 118 | 693 | 17.02742 | 8.696532 | 4.513217 |
| LEONE RD, 2020, NAT REV CANCER | 10.1038/s41568-020-0273-y | 2020 | 117 | 714 | 16.38655 | 11.80601 | 6.829202 |
